# Molecular Simulation-Elucidated Boronate Ester Gelation in Succinyl Chitosan-Based Hydrogels with Triple-Staged Thermoresponsive Sol–Gel–Sol Behavior

**DOI:** 10.1021/acs.biomac.6c00414

**Published:** 2026-06-22

**Authors:** Wen-Hsin Wang, Yue-Ci Wu, Hsiu-Min Hung, Kai-Chun Wang, Dhayanithi Senthilkumar, Ying-Chieh Hung, Chih-Yu Kuo

**Affiliations:** † Department of Chemical Engineering and Biotechnology, 34877 National Taipei University of Technology, Taipei City 10608, Taiwan; ‡ Institute of Biochemical and Biomedical Engineering, National Taipei University of Technology, Taipei City 10608, Taiwan; § International Graduate Program of Energy and Optoelectronic Materials Program (EOMP), National Taipei University of Technology, Taipei City 10608, Taiwan; ∥ High-value Biomaterials Research and Commercialization Center, National Taipei University of Technology, Taipei City 10608, Taiwan

## Abstract

A new class of triple-staged, thermosensitive hydrogels has been developed using double cross-linking between succinyl chitosan (SC) and poly­(*N*-isopropylacrylamide-*co*-5-methacrylamido-1,2-benzoxaborole) and poly­(NIPAM-*co*-MAAmBO) copolymers. Poly­(NIPAM-*co*-MAAmBO) was first synthesized via RAFT polymerization with various monomer ratios and cross-linked by the hydroxyl groups on chitosan with poly­(MAAmBO) segments to form the boronate ester bonding. Combining with the thermoresponsive poly­(NIPAM) segments, we successfully prepared a dual-cross-linked hydrogel demonstrating a unique triple-staged, temperature-induced, sol–gel–sol transition behavior. This hydrogel exhibits injectability and self-healing capabilities. The mechanical properties of hydrogels can be tuned by adjusting the copolymer structure, hydrogel composition, temperature, and pH conditions. Among the various formulations, the hydrogel with a composition of SC/poly­(NIPAM_90_-*co*-MAAmBO_10_) = 5/5 (w/w) exhibited superior mechanical properties, with a storage modulus (*G*′) of approximately 1000 Pa at pH 7.4 and 37 °C. Rheological analysis confirmed the stability, pH responsiveness, and reversible temperature-induced sol–gel–sol transitions of the hydrogel. In summary, we demonstrated that the microstructure and properties of the SC/poly­(NIPAM-*co*-MAAmBO) hydrogels could be tailored through dynamic boronate ester bonds and temperature-responsive hydrogen bonds, showcasing significant potential for biomedical applications.

## Graphical abstract

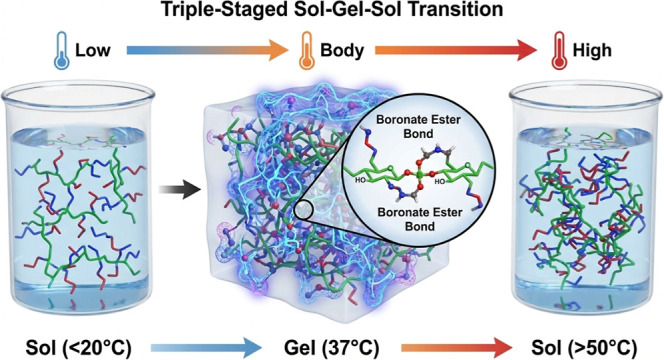

## Introduction

Hydrogels are three-dimensional cross-linked polymer networks with high water content and excellent tissue-like characteristics, making them attractive for a wide range of applications, including controlled release,^1,2^ drug delivery,^3,4^ tissue engineering,^5,6^ artificial cartilage,^7^ and bioelectronics.^8^ Depending on the cross-linking mechanism, hydrogels are generally classified as physically cross-linked or chemically cross-linked systems.^9^ Physically cross-linked hydrogels are often reversible and processable, but they typically suffer from limited mechanical stability. In contrast, chemically cross-linked hydrogels usually provide improved structural robustness, yet their irreversible networks often compromise shear-thinning behavior, adaptability, and self-repair capability.^10^ These trade-offs motivate the development of hydrogel networks that combine mechanical integrity with dynamic and stimulus-responsive behavior.

Dynamic covalent chemistry has emerged as an effective strategy to bridge this gap because it can provide the stability of covalent bonds while retaining reversible bond exchange and stress relaxation.^10,11^ Among dynamic covalent interactions, boronate ester bonds are particularly attractive for hydrogel construction because they can form under mild aqueous conditions without added catalysts and exhibit reversible binding with *cis*-diols.^12,13^ Their formation, however, is strongly governed by the p*K*_a_ of the boronic acid moiety and the solution pH.^14,15^ Conventional phenylboronic acids (p*K*_a_ = 8–9) generally require alkaline conditions for stable boronate ester formation, which limits their use in physiological or acidic environments.^16^ Benzoxaborole, a cyclic boronic acid derivative with a lower p*K*_a_ (∼7.2), offers improved diol affinity and enables more stable boronate ester formation near neutral pH.^17–20^ This feature makes benzoxaborole a promising building block for dynamic hydrogel networks intended for biomedical applications.

Apart from boronate ester dynamic covalent bonds, the temperature responsiveness of poly­(*N*-isopropylacrylamide) (PNIPAM) is another widely employed noncovalent cross-linking for hydrogel preparation. PNIPAM is composed of hydrophilic amide (−CONH−) groups and hydrophobic isopropyl (−CH­(CH_3_)_2_) side chains.^21–23^ When the environmental temperature exceeds its cloud point temperature (lower critical solution temperature, LCST), the interactions between isopropyl side chains are strengthened, while the interactions between the amide groups and water are weakened, leading to the aggregation of PNIPAM molecules. Consequently, PNIPAM hydrogels remain in a sol state at room temperature but undergo a cross-linking transition to a gel state upon contact with the human body.^19^ They exhibit in situ gelation properties. Therefore, the nonbiodegradable nature limits its application in biomedicine. To address this, many studies have introduced natural polymers into PNIPAM hydrogels, expanding their potential for broader biomedical applications.^24,25^

Despite these advantages, two important gaps remain in the current literature. First, most boronate ester hydrogel studies primarily emphasize formulation and bulk performance (e.g., gelation, self-healing, responsiveness). In contrast, the molecular mechanism of boronate ester formation in polysaccharide-based hydrogel environments remains poorly understood. In particular, the relative roles of different coordinating groups (e.g., hydroxyl vs amine functionalities) and their consequences for gelation efficiency, pH sensitivity, and rheological behavior are seldom clarified at the molecular level. Second, although thermoresponsive polymers have been extensively integrated into dynamic hydrogels, mechanistically understood carbohydrate-derived systems that exhibit programmable multistage thermal phase transitions (rather than a simple sol–gel switch) are still rare. Addressing these gaps would not only deepen fundamental understanding of dynamic polysaccharide networks but also enable more predictive structure-property design for injectable and stimuli-responsive biomaterials.

Herein, we report a dual-cross-linked, multiresponsive hydrogel platform constructed from succinyl chitosan (SC) and a RAFT-synthesized poly­(*N*-isopropylacrylamide-*co*-5-methacrylamido-1,2-benzoxaborole) copolymer (poly­(NIPAM-*co*-MAAmBO), denoted as PNB). In this design, dynamic boronate ester bonds between SC and benzoxaborole units are coupled with thermoresponsive PolyNIPAM interactions to generate an SC/PNB hydrogel with an unusual triple-staged temperature-induced sol–gel–sol transition. We systematically investigate the effects of copolymer composition, SC/PNB ratio, solid content, temperature, and pH on gelation and viscoelastic properties and evaluate the self-healing and injectability of the resulting hydrogels. In addition, density functional theory (DFT) calculations are employed to elucidate the boronate ester formation pathway and to connect molecular-level reaction energetics with the observed macroscopic gelation behavior. This study provides a mechanistically informed strategy for designing polysaccharide-based, dynamically cross-linked, thermoresponsive hydrogels with tunable properties for advanced biomedical applications.

## Experiment and Theoretical Modeling

### Materials

Succinic anhydride (SA), *N*-isopropylacrylamide (NIPAM), 5-Amino-2-(hydroxymethyl)­benzeneboronic acid dehydrate hydrochloride, 2-(dodecylthiocarbonothioylthio)-2-methylpropionic acid (DDMAT), and chitosan (medium molecular weight, degree of deacetylation >85%) (CS) were purchased from Angene, Merck, and Fluorochem, respectively. Thermo Scientific. Methacryloyl chloride and 4,4′-Azobis­(4-cyanovaleric acid) (ACVA) were purchased from Alfa Aesar. Organic solvents were obtained from Fisher Chemical. All chemical reagents are used directly as received without further purification.

### Molecular Weight and Distribution Determination of Chitosan

The molecular weight and distribution of chitosan were determined by high-performance size exclusion chromatography, using pullulan standards.^26^ The chitosan solution was prepared as 0.5 wt % in 0.2 M acetic acid, 0.1 M sodium acetate, and 0.008 M sodium azide and stirred for 24 h until fully dissolved. Data were acquired using SISC 3.2 integration software (Shun Hwa, EMB50S). The number-average molecular weight (*M*_n_), weight-average molecular weight (*M*_w_), and polydispersity index (PDI = *M*_w_/*M*_n_) were calculated as 205,320 ± 61,065 g/mol, 646,653 ± 63,240 g/mol, and 3.15 ± 0.69, respectively.

### RAFT Polymerization of Poly­(NIPAM-*co*-MAAmBO)

Methacrylamide benzoxaborole (MAAmBO) was synthesized as reported with modification.^27,28^ As shown in Table 1, various amounts of NIPAM, MAAmBO, DDMAT, and ACVA were fully dissolved in 5 mL DMF and reacted under a N_2_ atmosphere at 70 °C for 24 h. After cooling the reaction, the mixture was added dropwise into the cold ether to precipitate the product, poly­(NIPAM-*co*-MAAmBO). Poly­(NIPAM-*co*-MAAmBO) was recorded as PNB_10_ and PNF_5_, in which 10 and 5 indicate the molar percent of MAAmBO feeding content.

**Table 1:** Recipe for the Synthesis of Poly­(NIPAM-*co*-MAAmBO), PNBs

sample	NIPAM (mg)	MAAmBO (mg)	DDMAT (mg)	ACVA (mg)	DMF (mL)
PNB_10_	985	210	11.3	3.4	5
PNB_5_	1040	105	11.3	3.4	5

### Formation of SC/PNB Hydrogels

SC was prepared according to the previously reported method.^24^ First, SC was uniformly dissolved in PBS at pH 7.4 to create 10 and 7.5 wt % solutions. PNB was dissolved separately in PBS (pH 7.4) at 4 °C, and then, under low-temperature conditions, the PNB solution was mixed with the SC solution in various volume ratios of 10/0, 7/3, 5/5, 3/7, and 0/10 to form the SC/PNB solutions. After equilibrating the solutions by adjusting the temperature, the changes were observed, and gelation properties were assessed using the inverted vial test.

### Structural Characterizations of PNB Copolymers

The structure and composition information on MAAmBO and PNBs polymers was determined by NMR spectra using DMSO-*d*_6_ as the solvent. Molecular weight and distribution of PNB polymers were determined by gel permeation chromatography (GPC) using 5 wt % lithium chloride 1-methyl-2-pyrrolidone (NMP) solution as the eluent. Weight- and number-average molecular weights (*M*_w_ and *M*_n_, respectively) and molecular weight distribution were determined based on poly­(styrene) (PS) standards. FT-IR absorption spectra were measured at frequencies ranging from 4000 to 400 cm^–1^ with 4 cm^–1^ resolution. The samples were mixed with KBr and prepared as pellets. The PNB polymers were dissolved in PBS (pH = 5.5 and 7.4) or pH = 10.0 buffer as a 0.1 wt % solution. The transmittance of the solution at 480 nm was measured by a UV/vis spectrophotometer with a heating rate of 40 °C/min at different temperatures. The cloud point temperature of PNB polymers was decided as the drop point of the transmittance curve.

### Rheological Analysis of SC/PNF Hydrogels

Rheological behaviors of SC/PNB hydrogels were performed by a rheometer (Anton Paar, MCR 302) at constant strain (1%) and frequency (1 Hz) from 10 to 60 °C with a heating rate of 3 °C/min. Frequency scanning was conducted from 1 to 100 Hz at 37 °C and 0.1% strain. Strain sweep scanning tests were performed ranging from 0.1% to 1000% at a fixed frequency of 1 Hz. Cyclic temperature sweeping of SC/PNB hydrogels was conducted at 1% strain and 1 Hz frequency under 10 or 37 °C. The self-healing behavior was monitored by dynamic strain measurement of SC/PNB hydrogels under the alternative strain (γ = 0.1% or 1000%) at 37 °C.

### Stimuli-Responsive Properties of SC/PNB Hydrogels

SC/PNB hydrogels with various compositions were prepared and stored overnight at 4 °C to reach equilibrium. Temperature-induced sol–gel–sol transition behaviors were observed through the inverted vial tests under 4, 20, 37, and 60 °C. The self-healing behaviors of SC/PNB hydrogels were studied through visible examination at 37 °C. Disc-shaped hydrogels were fabricated and stained with different food dyes. Stained hydrogels were cut in half and placed touching each other. Images were taken at various intervals to observe the dye diffusion and healing process. The injectability of SC/PNB hydrogels was determined by the extrusion method. SC/PNB hydrogels were prepared in a 3 mL syringe and extruded from a 22G needle to write characters as NTUT. Images were taken to evaluate the clarity and continuity of the characters. The pH-responsive degradation of SC/PNB hydrogels was determined under pH = 7.4 or 5.5. SC/PNB hydrogels were prepared in syringes and incubated at 37 °C for 24 h to achieve complete gelation. After gel formation, each sample was weighed to obtain its initial weight (*W*_*i*_) and subsequently transferred to a 24-well culture plate. Buffer solutions at pH 7.4 or 5.5 were then added to the respective wells. The hydrogels were taken out at predetermined time intervals, gently blotted with lens-cleaning tissue to eliminate excess surface liquid, and weighed to determine the final weight (*W*_r_). The percentage of weight remaining was calculated using the following equation:

$$hydrolytic\;mass\;loss\;\left(\%\right)=\frac{W_{i}-W_{r}}{W_{i}}\times 100\%$$

All of the aforementioned processes must be operated at 37 °C.

### Theoretical Investigation

In this study, density functional theory (DFT) calculations were employed to investigate the cross-linking reaction mechanism of the SC/PNB hydrogel formation. The electronic potential diagram and corresponding activation barriers were determined by using DFT calculations performed with Gaussian 16.^29^ Geometry optimizations and frequency analyses of the reactants, products, and transition states were carried out with the long-range corrected B3LYP functional (CAM-B3LYP)^30^ method at a basis set of the 6–31G­(d,p) level. The empirical dispersion correlation (GD3)^31^ was combined with the CAM-B3LYP method. The optimized reactant and product structures exhibited no imaginary frequencies, confirming their identification as true minima on the potential energy surface; in contrast, the transition state structure displayed a single imaginary frequency corresponding to the reaction coordinate.

## Results and Discussion

### Characterization of SC and PNB Copolymers

As illustrated in Figure 1A, poly­(NIPAM-*co*-MAAmBO) (PNB) copolymers were synthesized using RAFT polymerization with NIPAM and MAAmBO as monomers. Figure 1B and Table 2 display the GPC analysis and results of PNB_10_ and PNB_5_. PNB_10_ exhibited a molecular weight of 31,628 with a PDI of 1.14, while PNB5 had a molecular weight of 30,780 and a PDI of 1.13. The molecular weights of PNB10 and PNB5 are slightly lower than the theoretical ones, which might be because of the reactivity of different monomers. On the other hand, the repeating-unit ratio of NIPAM and MAAmBO on the copolymer chain was determined by comparing the integration areas of the absorption peaks at 4.92 and 3.82 ppm, which correspond to the five-membered ester ring of MAAmBO and the isopropyl group of NIPAM, respectively. As shown in Table 2, the NIPAM/MAAmBO ratios for PNB_10_ and PNB_5_ were 90/10 and 94.9/5.1, respectively, indicating that RAFT polymerization enables precise control over the copolymer composition and molecular weight distribution. Additionally, PNIPAM, PNB_10_, and PNB_5_ were dissolved in a pH 7.4 buffer solution, and the changes in transmittance at 480 nm were recorded at different temperatures to determine their cloud point temperature. As depicted in Figure 1D, the cloud point temperature of pure PNIPAM was 32 °C. With an increase in MAAmBO content, the cloud point temperature of the copolymer gradually decreased. When 5 mol % MAAmBO was incorporated, the cloud point temperature of PNB_5_ was 28 °C; when 10 mol % MAAmBO was added, the cloud point temperature of PNB_10_ decreased to 26 °C. This phenomenon can be attributed to the hydrophobic nature of the MAAmBO monomer—an increase in its proportion renders the copolymer more hydrophobic. As the temperature rises, not only does the interaction between the isopropyl side chains in the copolymer intensify, but also the greater the amount of hydrophobic MAAmBO, the more the cloud point temperature shifts from 32 to 26 °C. The phase-transition behavior of PNB copolymers also exhibited pH dependence. The pH-dependent transmittance curves further demonstrate that the thermoresponsive behavior of PNB copolymers is strongly influenced by the ionization state of the benzoxaborole moieties, as shown in Figure S1. At pH 10.0, both PNB5 and PNB10 maintained high transmittance over the measured temperature range, suggesting that the increased ionization of benzoxaborole groups enhances copolymer hydrophilicity and suppresses PNIPAM-driven thermal aggregation. In contrast, clear phase transitions were observed at pH 7.4 and pH 5.5, where reduced ionization of the benzoxaborole groups decreases polymer–water affinity and facilitates dehydration and aggregation of the PNIPAM segments. Compared with PNB_10_, PNB_5_ showed a more apparent pH-dependent shift in cloud point temperature, likely because its thermal transition remains more strongly governed by PNIPAM hydration/dehydration. In PNB10, the higher benzoxaborole content already promotes stronger hydrophobic association and lowers the transition temperature, thereby reducing the apparent difference between pH 7.4 and pH 5.5. These results indicate that the phase-transition behavior of free PNB copolymers is governed by the combined effects of PNIPAM dehydration, benzoxaborole hydrophobicity, and pH-dependent ionization.

**Figure 1:**
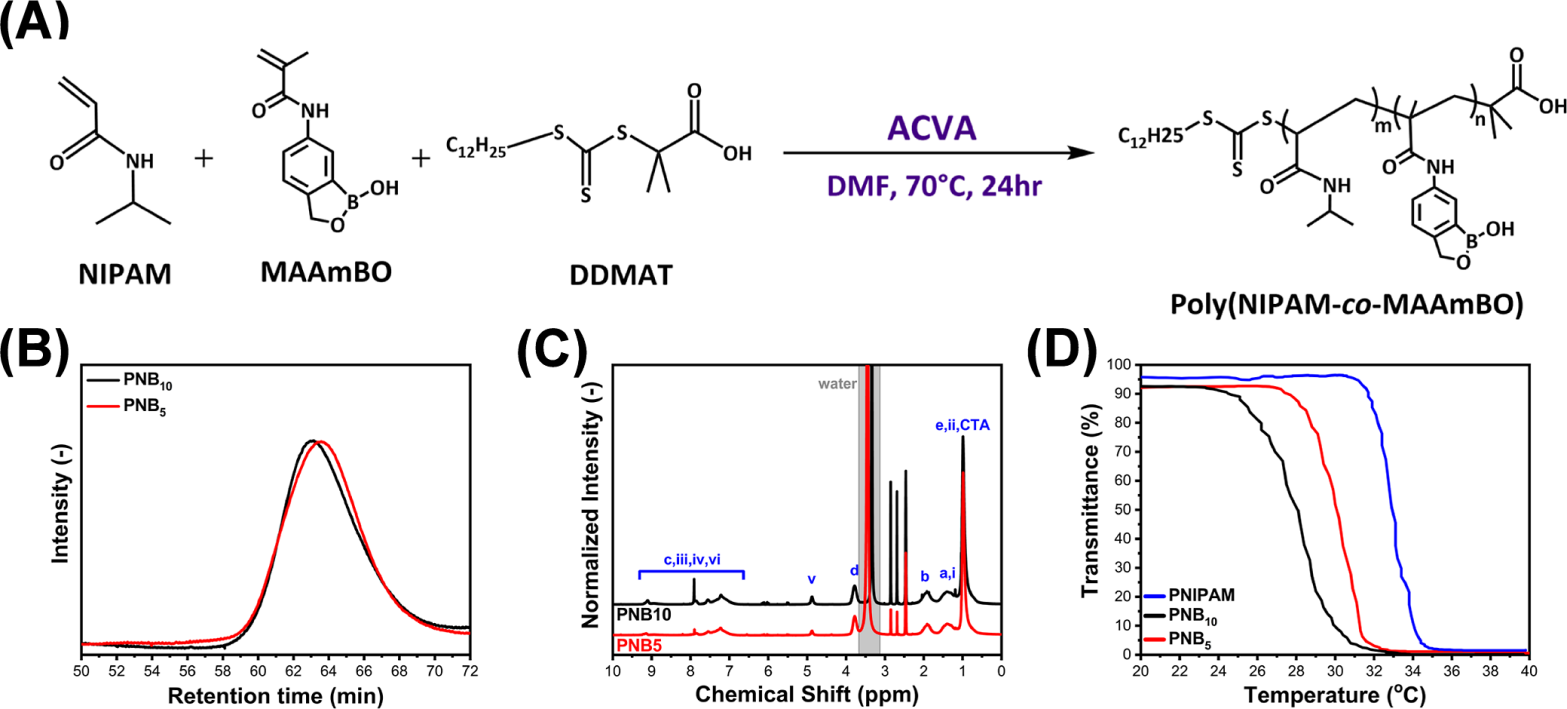
Characterization of as-synthesized, benzoxaborole-contained copolymers. (A) The mechanism of poly­(NIPAM-*co*-MAAmBO) synthesis via RAFT polymerization; (B) GPC, (C) NMR, and (D) phase transition temperature analyses of PNIPAM and PNBs at pH = 7.4.

**Table 2:** Molecular Weight and Distribution of Poly­(NIPAM-*co*-MAAmBO) by GPC and NMR Analyses

sample	(NIPAM/MAAmBO)^Feed^	(NIPAM/MAAmBO)^NMR^	Mn	Mn^theo^	PDI
PNB_10_	90/10	90.0/10.0	31,628	39,022	1.14
PNB_5_	95/5	94.9/5.1	30,780	37,698	1.13

### Fabrication and Properties of the SC/PNB Hydrogels (Morphology)

Figure 2A illustrates the mechanism of SC/PNB hydrogel preparation, where a 3D cross-linked network is formed through the dynamic covalent boronate ester bonds between the benzoxaborole groups on PNB copolymers and the hydroxyl groups on SC. Various weight ratios of SC/PNB were mixed to prepare and optimize the hydrogel composition, as shown in Figure 2B,C. It is known that higher molecular weight polymers generally promote gel formation through enhanced chain entanglement and improved three-dimensional network formation.^32^ In contrast, broader polydispersity may lead to heterogeneous network structures, thereby influencing the overall gelation behavior and stability of the hydrogel system.^33^ However, since both PNB10 and PNB5 possessed similar molecular weights and narrow PDIs, the observed differences in gelation and rheological behavior are mainly attributed to the benzoxaborole content and resulting cross-linking density rather than molecular weight distribution effects. It is obvious that both SC and PNB dissolved in a pH 7.4 buffer solution remained as liquid at all temperatures. Neither of them is able to form a gel independently. When SC and PNB were mixed at a total solid content of 7.5 wt %, the viscosity of the sample increased due to the formation of dynamic covalent boronate ester bonds. However, no gelation was observed at 4 or 20 °C in any composition. Upon heating the samples to 37 °C, gelation occurred in the SC/PNB = 7/3 and 5/5 mixtures. This was attributed to the formation of intermolecular hydrogen bonds in PNIPAM combined with the boronate ester bonds, which assisted in the hydrogel formation. Furthermore, it was visually evident that the SC/PNB = 5/5 hydrogel exhibited more solidification than the SC/PNB = 7/3 hydrogel, likely due to the higher amount of benzoxaborole in SC/PNB = 5/5, which allowed for more cross-linking with hydroxyl groups in SC, associated with the dehydration-induced aggregation of PNIPAM at elevated temperatures, leading to increased cross-linking density. Interestingly, when the temperature was further increased, the SC/PNB = 7/3 and 5/5 samples transitioned back to a sol state. This phenomenon resulted from the interplay between the intermolecular hydrogen bonds of the PNIPAM chains and the dynamic covalent boronate ester bonds. However, for SC/PNB = 3/7 samples, no gelation was observed under the test conditions. It is the insufficient amount of boronate ester bonds owing to the lack of SC ratio that hindered the gel formation. We will further discuss the mechanism using rheological analysis. Additionally, when SC/PNB was prepared in alkaline (pH = 10.0) or acidic (pH = 4.0) solutions, similar sol–gel–sol transitions were observed as in neutral environments (Figure 3), though the mechanical strength of the hydrogels varied slightly. As aforementioned, SC/PNB hydrogels are able to form at not only neutral pH but also acidic conditions due to the introduction of benzoxaborole groups. FTIR spectroscopy was also employed to analyze the structural changes in SC, PNB, and SC/PNB hydrogels. As shown in Figure 2D, the FTIR spectrum of PNB displays characteristic peaks at 3288 cm^–1^ for N–H stretching, 1533 cm^–1^ for N–H bending, 1647 cm^–1^ for C=O stretching vibrations, and 1387 cm^–1^ and 1366 cm^–1^ for symmetric isopropyl methyl bending vibrations. After forming the hydrogel with SC, the B–O–C signal at 1280 cm^–1^ was slightly diminished, likely due to the condensation of the boronate ester structure in MAAmBO with the hydroxyl groups on SC, resulting in the formation of dynamic covalent boronate ester bonds.

**Figure 2:**
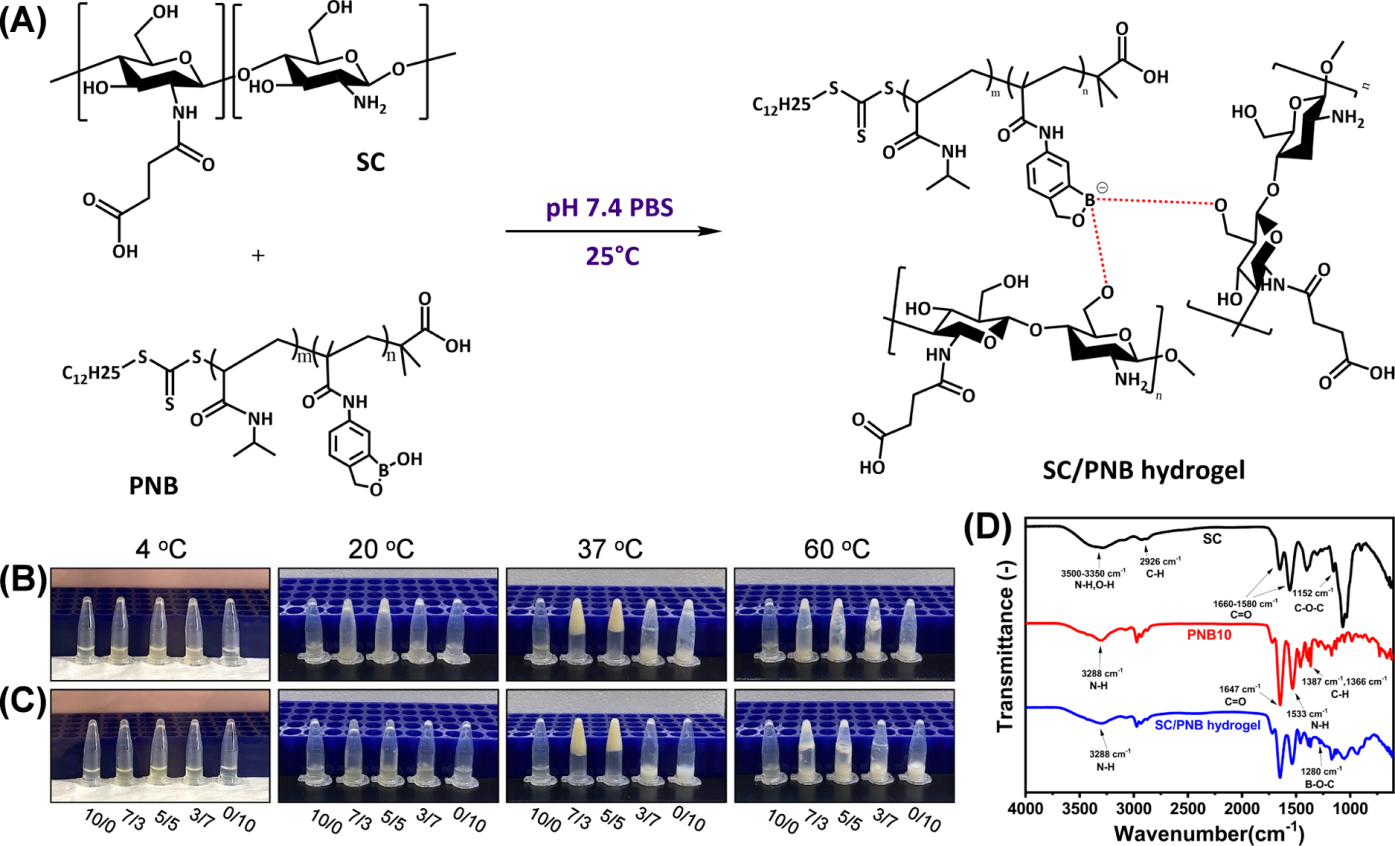
Preparation and characterization of the SC/PNB hydrogel. (A) Schematic illustrations of the preparation process of SC, the PNB copolymer, and the corresponding SC/PNB hydrogels. Optimization of SC/PNB hydrogel compositions at 7.5 wt % with various SC/PNF weight ratios at pH = 7.4: (B) SC/PNB_10_ and (C) SC/PNB_5_ under different temperatures. (D) FTIR analysis of SC, PNB polymers, and SC/PNB_10_ and SC/PNB_5_ hydrogels.

**Figure 3:**
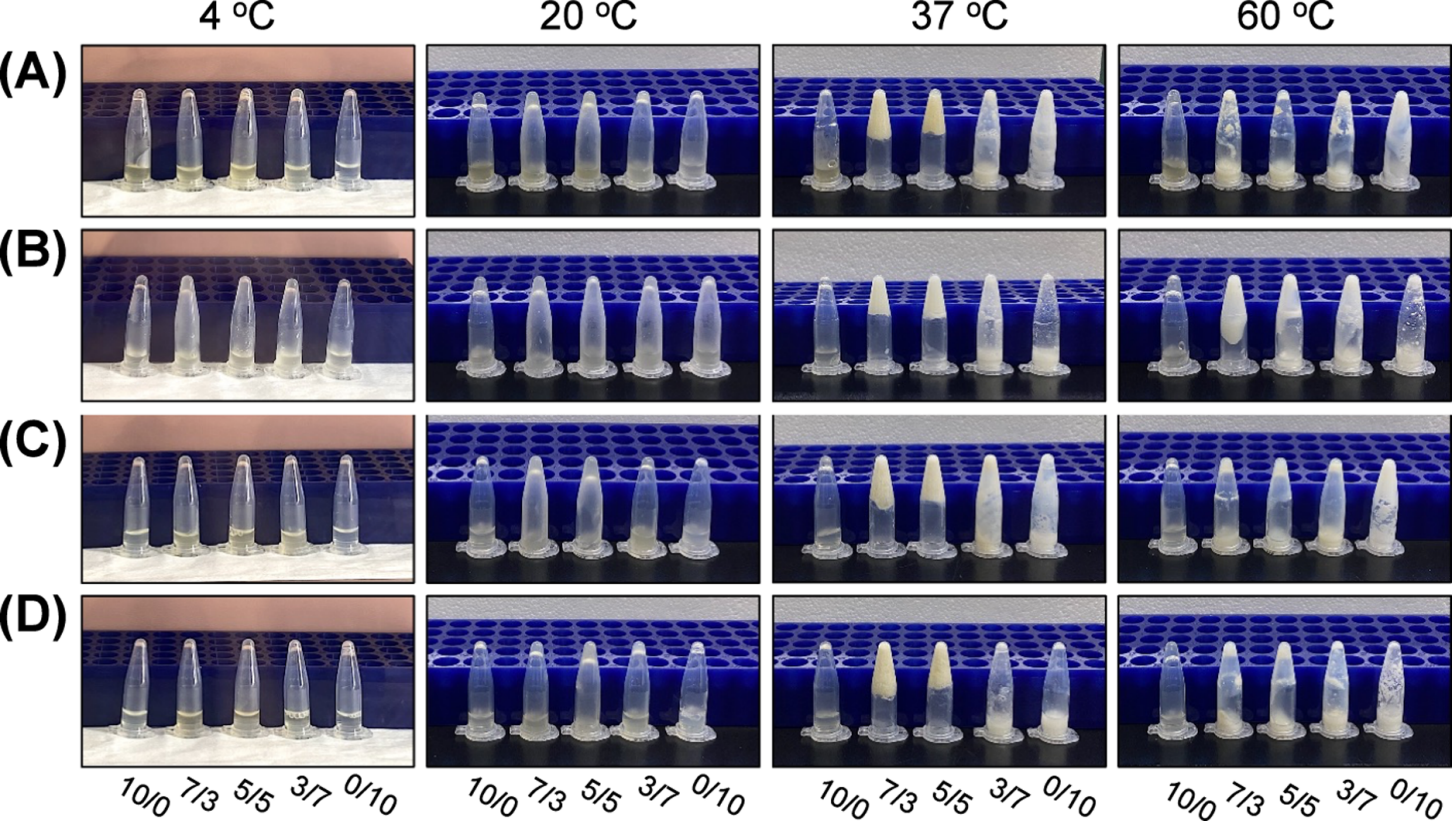
SC/PNB hydrogels at 7.5 wt % with various SC/PNB weight ratios under pH = 10: (A) SC/PNB_10_ and (B) SC/PNB_5_, and pH = 4.0: (C) SC/PNB_10_ and (D) SC/PNB_5_, at different temperatures.

### Temperature-Responsive Behaviors of SC/PNF Hydrogels

To investigate the effects of temperature, composition, and different copolymers on the mechanical properties of SC/PNB hydrogels, rheological measurements were conducted, examining the changes in storage modulus (*G*′) and loss modulus (*G*″) between 10 and 60 °C. When the total solid content was fixed at 7.5 wt %, it was observed that SC, PNB_10_, and PNB_5_ exhibited excellent fluidity across different temperatures (Figures 2B and 2C). As a result, the rheological analysis consistently showed *G*″ > *G*′, indicating a sol state, with modulus values below 50 Pa. However, upon the mixing of SC and PNB copolymers, the SC/PNB hydrogels displayed distinct temperature-responsive behaviors. Regardless of the SC/PNB ratios (7/3, 5/5, or 3/7), the modulus of SC/PNB initially increased and then decreased with rising temperature as depicted in Figure 4A. The sol–gel–sol, triple-staged transition was attributed to a synergistic effect between the boronate ester bonds and the hydrogen bonding of PNIPAM. At low temperatures, SC/PNB is primarily stabilized by the boronate ester bonds in a solution state with *G*″ > *G*′. As the temperature increases, the hydrogen bonds between the PNIPAM chains and water molecules are disrupted by thermal energy in which the inner molecular forces between PNIPAM chains exceed the interactions with water, leading to the aggregation of polymers. The phenomenon enhanced the mechanical properties and stabilized the hydrogel structure of SC/PNB, shifting it to a gel state with *G*′ > *G*′′. However, at higher temperatures, boronate ester bonds became less stable, and when the temperature reached nearly 50 °C, the internal bonds of the SC/PNB hydrogel broke down, leading to the third phase of gel-to-sol transition.^34^ Comparing with the mechanical properties of different SC/PNB hydrogel compositions, it was noted that SC/PNB = 7/3 exhibited weaker mechanical strength compared to SC/PNB = 5/5, while SC/PNB = 3/7 failed to form a gel (*G*′′ > *G*′). It likely arises from the higher proportion of benzoxaborole polymers in SC/PNB = 5/5 than SC/PNB = 7/5, allowing for the formation of more boronate ester bonds in the hydrogel. SC/PNB = 3/7 did not form a gel while showing a slight increase in mechanical strength with increasing temperature. This is due to an insufficient number of hydroxyl groups to create a stable 3D cross-linked network, given the lower chitosan content, as shown in the red curve in Figures 4A and 4B. On the other hand, a comparison between SC/PNB hydrogels prepared with PNB_10_ and PNB_5_ exhibited similar trends as temperature-induced, triple-staged behaviors. SC/PNB_10_ hydrogels demonstrated superior mechanical properties to SC/PNB_5_ hydrogels, likely because PNB_10_ contained a higher percentage of benzoxaborole, which could form more cross-linking with the hydroxyl groups on chitosan. Additionally, the effect of different solid content on the mechanical strength of SC/PNB hydrogels was evaluated. Figures S2 and S3 show the gelation behavior and rheological analysis of SC/PNB_10_ and SC/PNB_5_ hydrogels at 10 wt %. As solid content increased, both SC/PNB = 7/3 and SC/PNB = 5/5 exhibited gel states (G′ > G″) across all temperatures for both SC/PNB_10_ and SC/PNB_5_ hydrogels. Interestingly, as shown by the blue curves in Figures S2 and S3, the storage modulus of SC/PNB_10_ = 7/3 and SC/PNB_10_ = 7/3 hydrogels sharply increases when the temperature is raised beyond 50 °C. This unexpected enhancement in mechanical properties is hypothesized to result from intermolecular hydrogen bonding within the PNIPAM chains,^35^ which leads to further contraction and aggregation, encapsulating the broken boronate ester bonds and reinforcing the hydrogel structure. In addition to the chain entanglements, forming a denser network at higher temperatures also contributed to the positive dependence of moduli, resulting from the thermal-induced water evaporation inside the hydrogels.^36^

**Figure 4:**
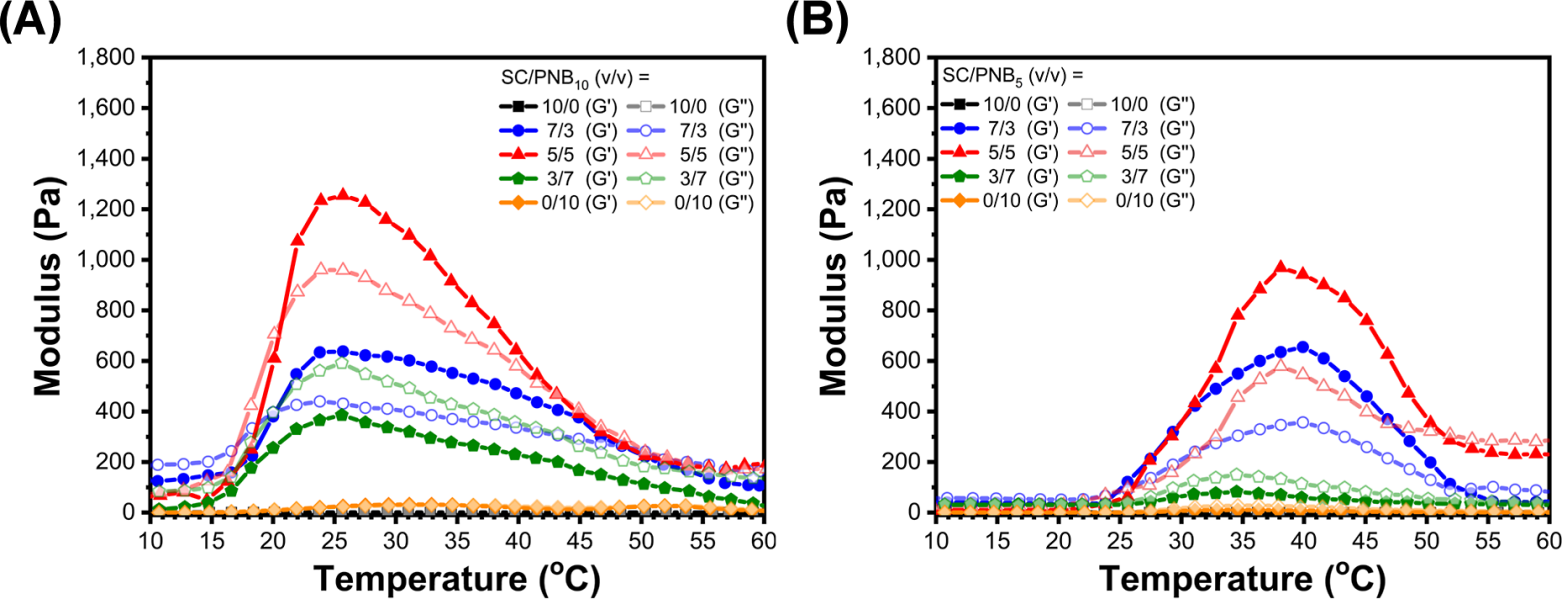
Rheology analysis for SC/PNB solutions at constant strain and frequency (γ = 1%, *f* = 1 Hz) from 10 to 60 °C with the heating rate as 3 °C/min: (A) SC/PNB_10__7.5 wt % and (B) SC/PNB_5__7.5 wt %.

Due to the formation of boronate ester bonds highly depending on the p*K*_a_ of benzoxaborole and the pH of the solution, SC/PNB = 5/5 was chosen to demonstrate the unique mechanical property changes at different temperatures under various pH conditions. Figure 5 illustrates that both SC/PNB_10_ = 5/5 (5A) and SC/PNB_5_ = 5/5 (5B) hydrogels exhibit a similar temperature-induced sol–gel–sol triple-staged transition under different pH conditions, with phase transitions occurring around 22 and 50 °C. Rheological analysis also revealed that the mechanical performance of the hydrogels is higher in acidic environments compared to alkaline conditions. This enhancement in mechanical strength in acidic environments can be attributed to the fact that SC has a lower p*K*_a_ than benzoxaborole, acting as a Lewis acid. In acidic conditions, the hydroxyl groups in SC are more likely to dissociate into O^–^, which can bind to the empty electron orbitals of boron in benzoxaborole, increasing the possibility of bond formation between SC and benzoxaborole.^20,37^ Consequently, the mechanical strength of the hydrogel is higher in acidic environments compared to alkaline ones.

**Figure 5:**
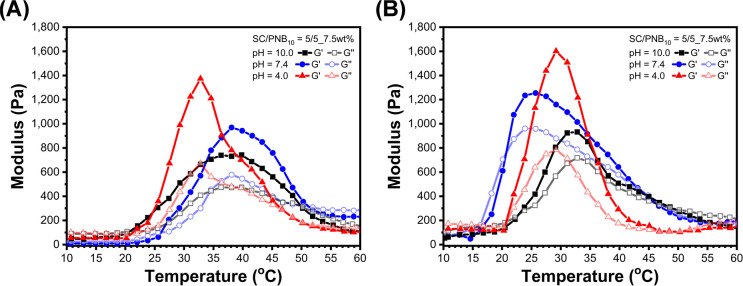
pH-dependent rheology analysis of SC/PNB_7.5 wt % solutions at constant strain and frequency (γ = 1%, *f* = 1 Hz) from 10 to 60 °C with the heating rate as 3 °C/min under pH = 10.0, 7.4, and 4.0: (A) SC/PNB_10__7.5 wt % and (B) SC/PNB_5__7.5 wt %.

SC/PNB hydrogels exhibited a notable temperature-induced, sol–gel–sol triple-staged phase transition, which involved the formation and dissociation of boronate ester bonds and hydrogen bonds at varying temperatures. Thus, rheological analyses were employed to investigate whether the morphology changes of SC/PNB hydrogels were reversible under specific temperature cycles. As shown in Figures 6A and 6B, both SC/PNB_10_ = 5/5 and SC/PNB_5_ = 5/5 hydrogels exhibit good dispersion at 10 °C (*G*′′ > *G*′), while gel formation occurs at 37 °C (*G*′ > *G*″). This behavior can be attributed to the fact that the SC/PNB composite materials only form boronate ester bonds at low temperatures. As the temperature rises, the PNIPAM chains generate intermolecular hydrogen bonds, leading to aggregation and gelation. Furthermore, due to the reversible temperature-responsive properties of the PNIPAM chains, the SC/PNB composites exhibit excellent reversibility in the sol–gel–sol three-phase transition when cycled between low and high temperatures. Neither storage modulus nor loss modulus was compromised by the number of temperature cycles. When the hydrogel composition was adjusted to SC/PNB = 7/3 (as shown in Figure S4), although the trend remained similar to SC/PNB = 5/5, the difference in mechanical strength between low and high temperatures was smaller, and the rate of sol–gel transition was slower. A lower PNB copolymer ratio in SC/PNB = 7/3 than in SC/PNB = 5/5 leads to the reduced cross-linking density, making the temperature response less pronounced. The sol state of SC/PNB hydrogel at low temperatures and its gelation at physiological temperatures, combined with the affinity of boronate ester bonds for cell membranes, make the SC/PNB hydrogel a promising candidate for 3D cell culture scaffolds. The SC/PNB solution and target cells could be mixed well under low temperatures while forming cell-embedded biomedical gel at physiological temperature spontaneously. The reversible sol–gel transitions offer promising applications in studying cell–cell interactions, making SC/PNB hydrogels a powerful tool for 3D cell culture research.

**Figure 6:**
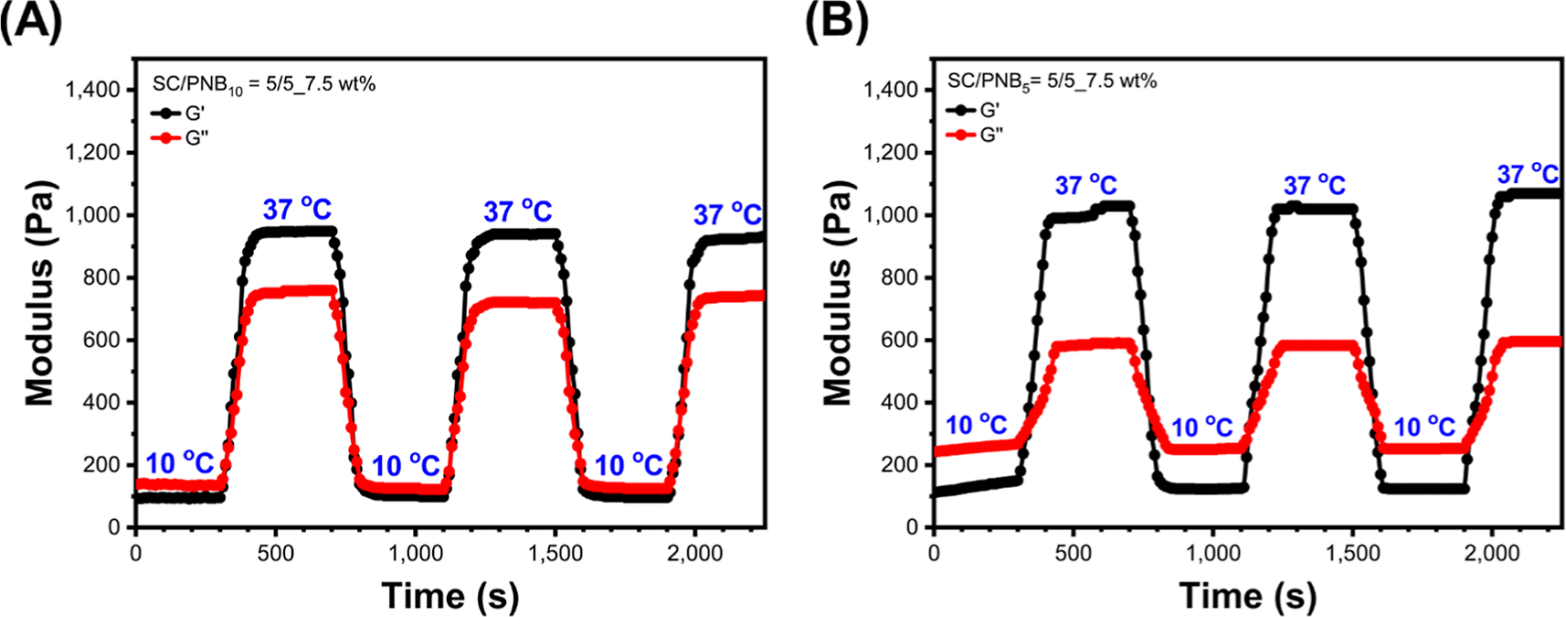
Cyclic temperature sweeping (10 or 37 °C) of SC/PNB = 5/5_7.5 wt % solutions at constant strain and frequency (γ = 1%, *f* = 1 Hz): (A) SC/PNB_10_ and (B) SC/PNB_5_.

### Self-Healing and Injectable Ability of SC/PNB Hydrogels

Due to the relatively low bond energy of boronate ester bonds, the SC/PNB hydrogel can be easily broken and reformed. The dynamic covalent bonding endows SC/PNB hydrogels with self-healing capabilities when subjected to repeated high and low strain cycles, a property that is highly beneficial for applications in drug delivery, tissue repair, and other biomedical fields. To investigate the self-healing properties of SC/PNB hydrogels, we analyzed the strain cycle tests for SC/PNB_10_ and SC/PNB_5_ hydrogels with compositions of 7/3 and 5/5 (solid content = 7.5 wt %). As shown in Figures 7A, 7B, and S5, under the synergistic effects of boronate ester and hydrogen bonds at 37 °C, the SC/PNB hydrogels exhibit a gel state (*G*′ > *G*″) under low strain but transition to a sol state (*G*″ > *G*′) under high strain. Hydrogels with a higher proportion of benzoxaborole (e.g., SC/PNB_10_ > SC/PNB_5_ or SC/PNB = 5/5 > 7/3) demonstrated superior self-healing abilities. Meanwhile, the self-healing capability of SC/PNB hydrogels was observed visually. Different colored hydrogels were recombined, and their self-healing behavior was observed at 37 °C after being stained with different colors and cut in half. The cut hydrogels began to self-heal immediately upon contact, and after 3 h, the hydrogels were fully healed. The distinct yellow-blue boundary gradually turned into a mixed green color, further confirming that SC/PNB hydrogels form a 3D cross-linked network through dynamic covalent bonds, as shown in Figure 7C,D. Finally, the fully healed hydrogels were lifted by tweezers so as to visually verify that the hydrogen and boronate ester bonds successfully reconnected the SC/PNB hydrogels. The phenomenon once again correlates with the findings from rheological analysis, providing clear evidence of the self-healing properties of the SC/PNB hydrogels. The hydrogel stability was additionally evaluated in PBS under different pH conditions (pH = 7.4 and 5.5). Figure S7 shows the mass loss behavior of SC/PNB hydrogels under physiological and mildly acidic conditions over 30 days. All hydrogels exhibited a relatively rapid initial mass loss during the first several days, followed by a slower erosion phase, which is characteristic of dynamic cross-linked hydrogels. The initial weight reduction is mainly attributed to the diffusion of loosely associated polymer chains and relaxation of reversible boronate ester networks upon water uptake. Subsequently, the remaining cross-linked structure maintained partial network integrity, resulting in a gradual stabilization of mass loss. Under pH 7.4 conditions, SC/PNB_10_ hydrogels exhibited lower mass loss than SC/PNB_5_ hydrogels, indicating that the higher benzoxaborole content increased the effective cross-linking density and improved network stability. In contrast, hydrogels incubated at pH = 5.5 showed higher mass loss overall, likely due to the reduced stability of boronate ester bonds and increased protonation-induced swelling of succinyl chitosan chains under acidic conditions. Interestingly, the difference between PNB_10_ and PNB_5_ became less pronounced at lower pH, suggesting that acidic environments partially weaken the stabilizing contribution of benzoxaborole-mediated dynamic covalent cross-linking.

**Figure 7:**
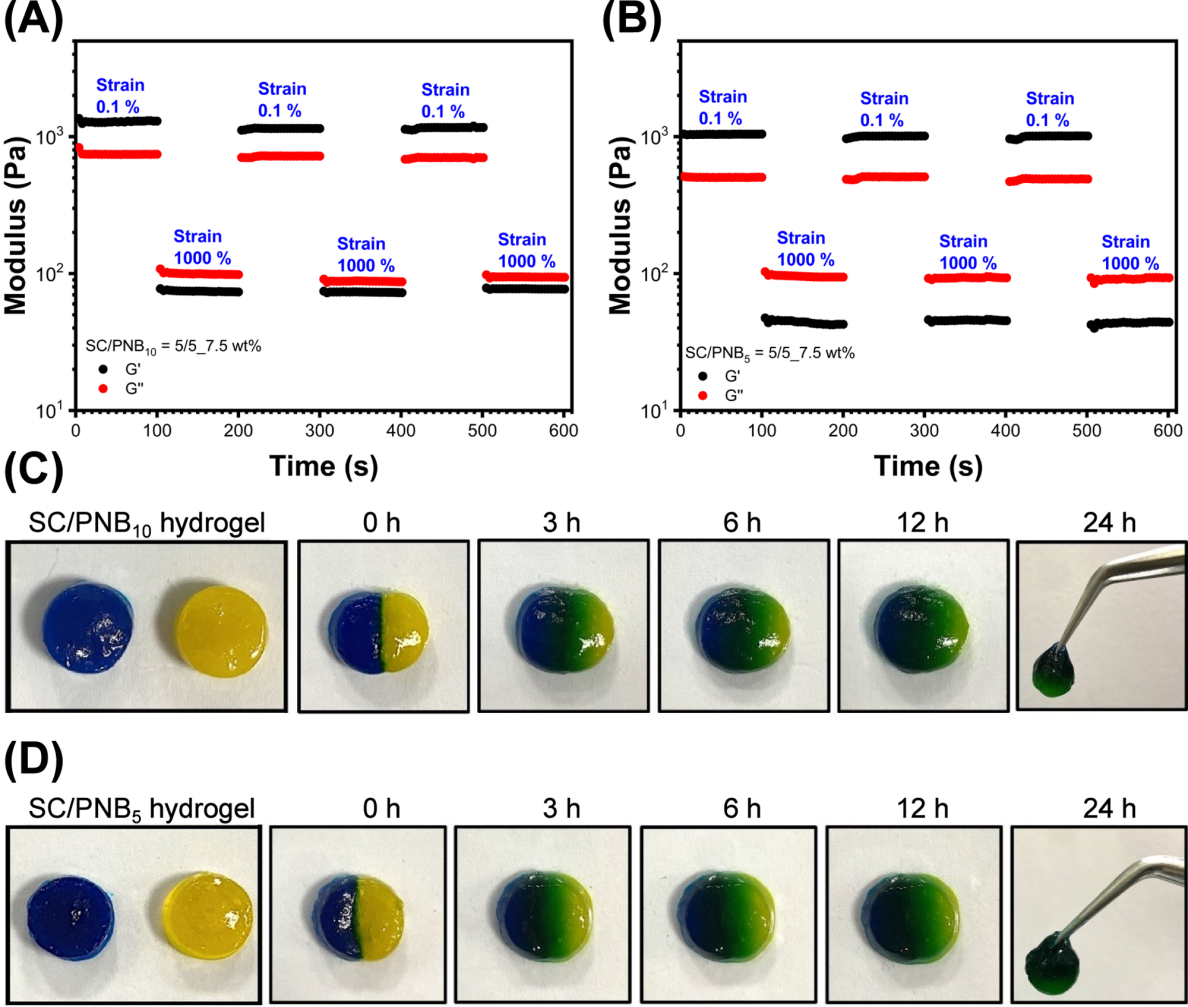
Self-healing behavior of SC/PNB = 5/5_7.5 wt % hydrogels at 37 °C. Dynamic strain measurement (γ = 0.1% or 1000%): (A) SC/PNB_10_ hydrogel and (B) SC/PNB_5_ hydrogel. Macroscopic self-healing images of (C) SC/PNB_10_ hydrogel and (D) SC/PNB_5_ hydrogel.

Storage modulus (*G*′) and loss modulus (*G*″) are critical parameters for characterizing the properties of hydrogels. They provide insights into the mechanical strength and viscoelastic behavior of hydrogels through rheological analysis under dynamic frequency and strain amplitude sweeps. Figure 8A illustrates the relationship between *G*′ and *G*″ with frequency for SC/PNB hydrogels, showing the viscoelastic behavior of the hydrogels containing different levels of benzoxaborole. It can be observed that for SC/PNB = 5/5, *G*′ consistently remains higher than *G*″, indicating a stable gel state. Both SC/PNB_10_ = 5/5 and SC/PNB_5_ = 5/5 exhibit excellent stability. As the frequency increases, the mechanical strength improves, likely due to heat generated during the measurement that strengthens intermolecular interactions in the PNIPAM segments. Figure 8B demonstrates the strain sweep tests for SC/PNB hydrogels at a fixed frequency of 1 Hz, with the strain range varying from 0.1% to 1000%. SC/PNB_5_ = 5/5 displays greater toughness due to fewer boronate ester bonds formed with SC, leading to a higher fracture strain (365%) compared to SC/PNB_10_ = 5/5 (233%). When the hydrogel composition is adjusted to SC/PNB = 7/3 (Figure S6A,B), the rheological analysis trends remain similar to SC/PNB = 5/5, but the mechanical strength is lower. The fracture strains for SC/PNB_10_ = 7/3 and SC/PNB_5_ = 7/3 are 147% and 325%, respectively. This further illustrates that the borate ester content is the key factor determining the cross-linking density and structural strength of the SC/PNB hydrogels.

**Figure 8:**
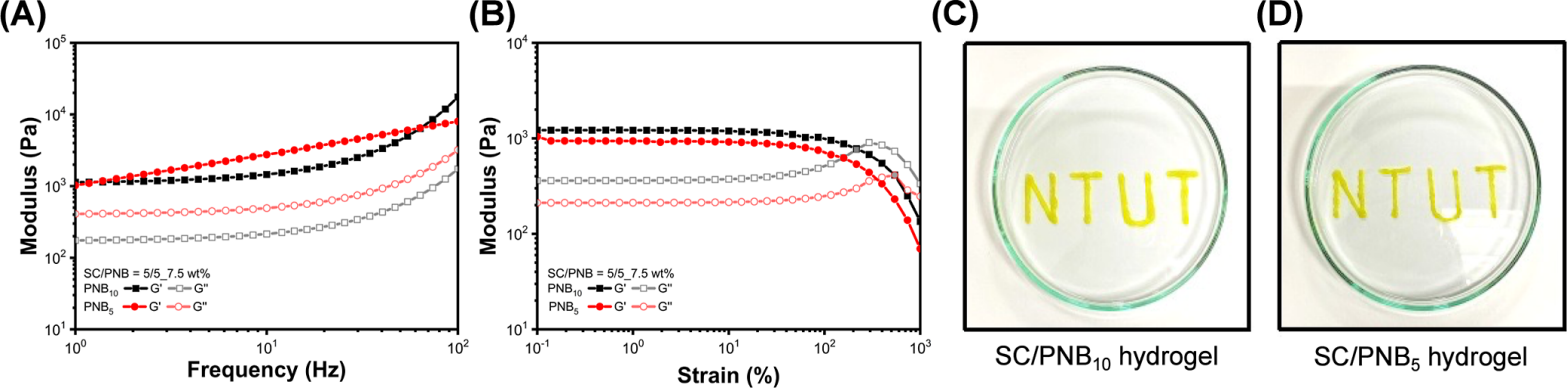
Rheology analysis for SC/PNB = 5/5_7.5 wt % hydrogels at 37 °C with PNB_10_ or PNB_5_. (A) Frequency sweeps from 1 to 100 Hz with a constant strain of 1% and (B) strain sweep scanning ranging from 0.1% to 1000% at a fixed frequency of 1 Hz. Injectability of SC/PNB = 5/5_7.5 wt % hydrogels: (C) SC/PNB_10__7.5 wt % hydrogel and (D) SC/PNB_5__7.5 wt % hydrogel.

Moreover, the strain sweep results show that SC/PNB hydrogels have high stability under low-strain conditions. The cyclic strain tests reveal the self-healing capabilities of these hydrogels, indicating that SC/PNB hydrogels possess injectable properties. Injectable hydrogels are crucial for drug delivery systems, as they allow for precise, localized treatment of affected areas through in situ release of therapeutic agents. To further explore this application, SC/PNB hydrogels with different PNB compositions were prepared, and the injectability was examined. As shown in Figures 8C,D and S6C,D, the SC/PNB hydrogels exhibit excellent injectability. When extruded through a syringe and used to write the English letters “NTUT”, the hydrogels produced smooth, intact, and continuous lines without any breakage, demonstrating their potential for in situ injectable drug release applications.

### Theoretical Evaluation of the SC/PNB Hydrogel Formation Mechanism

To model the local cross-linking chemistry of the SC/PNB hydrogel, a simplified molecular analogue was employed. The polymeric succinyl chitosan was represented by a single glucosamine unit bearing a vicinal diol, hydroxymethyl, and amino groups (Figure 9a), while phenylboronic acid was modeled using a truncated aromatic boronic acid fragment (Figure 9b). This approach preserves the essential hydrogen-bonding environment, boron Lewis acidity, and diol geometry for the investigation of the mechanism of boronate ester formation. Explicit water molecules were incorporated into the DFT framework to reliably capture proton transfer events during boronate ester formation.

**Figure 9:**
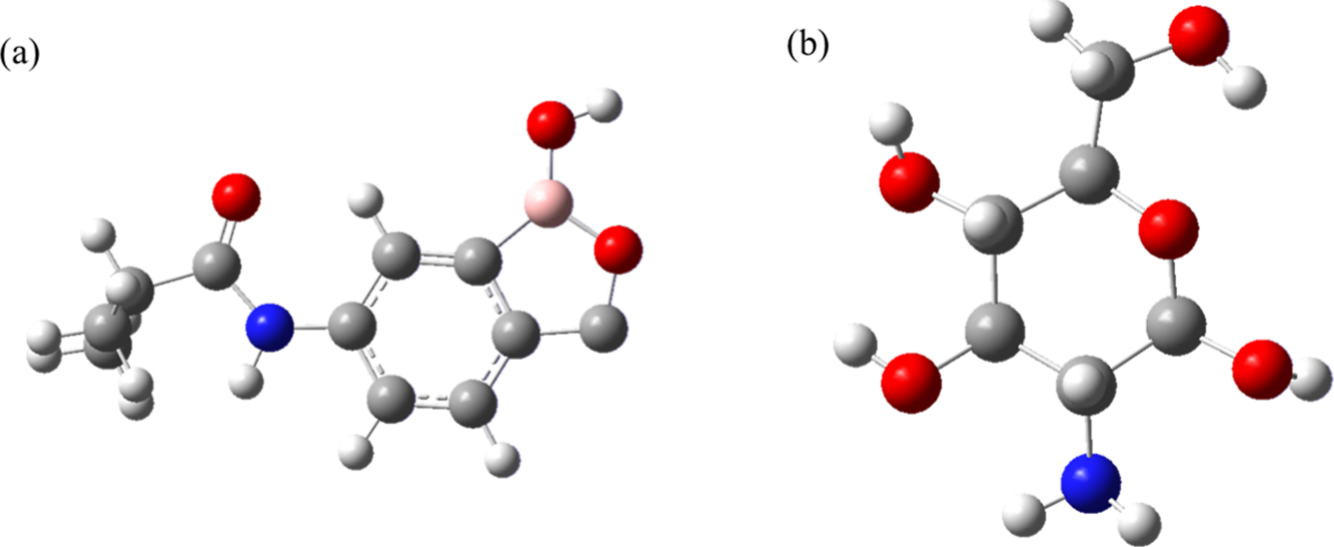
Optimized initial configurations used for DFT calculations. (a) A simplified aromatic boronic acid model to represent the reactive site of PNB. (b) A simplified SC model using an isolated glucosamine model to represent the reactive site of poly SC.

Two reaction pathways, each consisting of five elementary steps, for boronate ester formation involving a molecule containing both an amino group and a hydroxyl group were proposed and investigated. The simulated results, including the optimized structures of the five reaction steps and the corresponding electronic potential energy diagram along the reaction coordinate, are shown in Figures 10a and 10b, respectively. Basically, the local structural rearrangements during the nucleophilic reaction involving the hydroxyl and amino groups are similar. Therefore, the optimized snapshot of the boronic acid–hydroxyl group pathway is taken as a representative structure and is shown in Figure 10a. The electronic potential energy diagram corresponds to the boronic acid–hydroxyl group pathway, and boronic acid–amino groups are represented using O–H–B–O and H_2_N–B–O in Figure 10b, respectively. As shown in Figure 10a, in the initial configuration R0, the hydroxyl groups of boronic acid and the hydroxyl groups of SC are separated and stabilized by the formation of a hydrogen bond network with surrounding water molecules. Upon closer approach, configuration R1, the hydroxyl oxygen of the SC hydroxyl group begins to nucleophilically attack the boron center. Explicit water molecules rearrange to form a proton transfer pathway, preactivating the system for the forthcoming transition state. The third step is the transition state TS1, which corresponds to the highest-energy point along the reaction coordinate. In this configuration, the B–O­(diolate) bond is partially formed, while one B–OH bond is partially cleaved. Proton transfer is promoted from the SC hydroxyl group to the boronic acid hydroxyl group through the water HB network. Following TS1, the B–OH accepts a proton and forms a bound water molecule as shown in the configuration P1. In the final configuration P2, the boronic acid fully turns into a boronate ester with a discrete water molecule released. This product configuration underpins the dynamic yet robust cross-linking behavior observed in boronate ester hydrogels. Figure 10b summarizes the electronic potential energy profiles along the reaction pathway (R0–R1–TS1–P1–P2) for two coordination modes: the amine-assisted (H_2_N···B–O) and hydroxyl-assisted (H–O···B–O) pathways. From R0 to R1, the amine-assisted pathway exhibits a substantial energy increase of 57.4 kJ·mol^–1^, whereas the hydroxyl-assisted pathway requires only 5.36 kJ·mol^–1^, indicating that hydroxyl–boron interactions provide a significantly lower-energy preactivation route. During the proton-transfer activation step from R1 to TS1, the transition state is located at approximately 83–84 kJ·mol^–1^ for the amine-assisted pathway and 55–56 kJ·mol^–1^ for the hydroxyl-assisted pathway. The markedly lower activation barrier observed for the hydroxyl-assisted route highlights its kinetic advantage in promoting boronate ester formation under aqueous conditions. In the product formation stage (TS1 → P1 → P2), the final product energies differ substantially between the two pathways, with values of ∼23.8 kJ·mol^–1^ for the hydroxyl-assisted route and ∼60.0 kJ·mol^–1^ for the amine-assisted route. These results indicate that hydroxyl coordination not only lowers the activation energy but also yields a more thermodynamically stable boronate ester configuration. Overall, the DFT results demonstrate that TS1 constitutes the rate-limiting step in boronate ester formation and that hydroxyl-assisted coordination offers both kinetic and thermodynamic superiority over the amine-assisted pathway. The calculated results provide strong theoretical support for the proposed covalent boronate ester bond formation mechanism.

**Figure 10:**
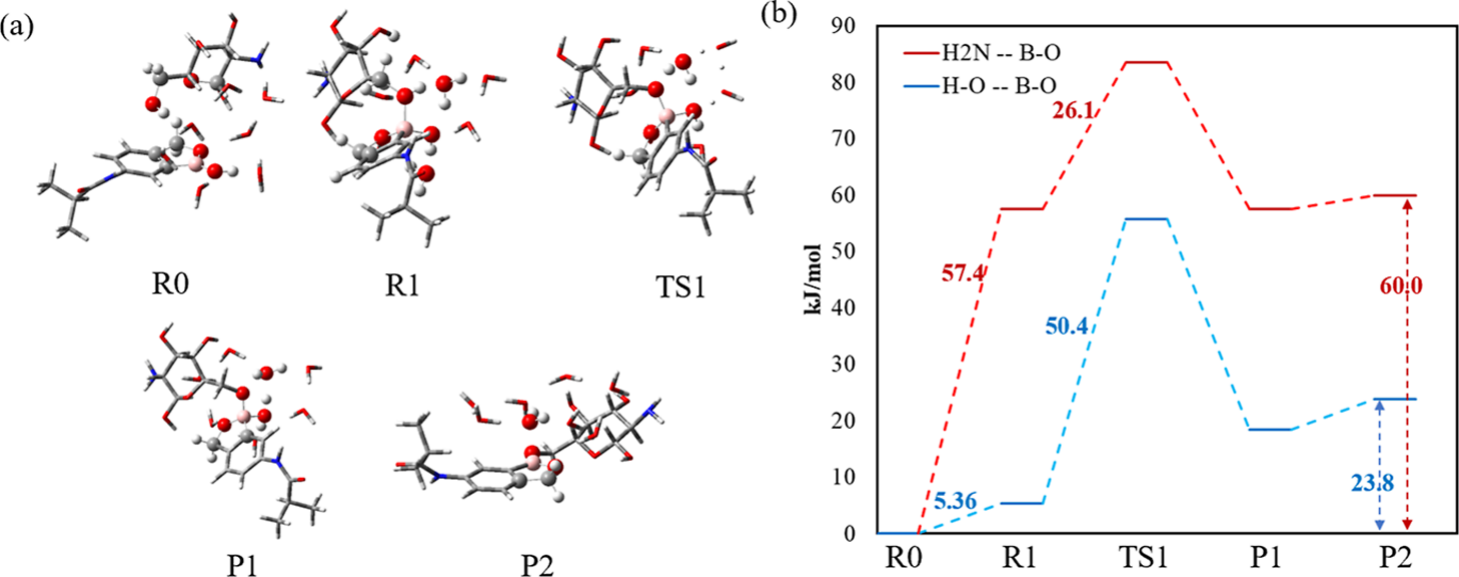
(a) Optimized geometries along the boronate ester formation pathway between boronic acid and the SC model, including the reactant complex (R0), preactivated intermediate (R1), transition state (TS1), metastable boronate intermediate (P1), and final boronate ester product with water release (P2). (b) Corresponding electronic potential energy diagram for two coordination pathways: amine-assisted (H_2_N···B–O, red) and hydroxyl-assisted (H–O···B–O, blue).

## Conclusion

In summary, we developed a dual-cross-linked, multiresponsive hydrogel platform by combining succinyl chitosan (SC) with RAFT-synthesized poly­(*N*-isopropylacrylamide-*co*-5-methacrylamido-1,2-benzoxaborole) (PNB). The integration of dynamic boronate ester cross-links and thermoresponsive PNIPAM interactions produced SC/PNB hydrogels with a distinctive triple-staged temperature-induced sol–gel–sol transition, together with self-healing, injectability, and composition-/pH-dependent viscoelastic tunability. Rheological measurements reveal that at low temperatures, the materials remain in a sol state, stabilized predominantly by dynamic boronate ester bonds. Upon heating to near-physiological temperatures, disruption of PNIPAM–water interactions and enhancement of intermolecular hydrogen bonding drive polymer aggregation, increase cross-linking density, and promote gel formation (*G*′ > *G*″). At higher temperatures, the boronate ester bonds become destabilized, leading to breakdown of the cross-linked network and reversion to a sol state, thus completing the triple-stage sol–gel–sol cycle. Critically, the incorporation of molecular simulation provides a mechanistic foundation for these macroscopic observations. DFT calculations using simplified SC and benzoxaborole models demonstrate that boronate ester formation is governed by a hydroxyl-assisted pathway that exhibits both a markedly lower activation barrier and a more favorable final-state energy than the amine-assisted route. The identification of the rate-limiting transition state, along with the clear kinetic and thermodynamic preference for hydroxyl coordination, rationalizes the efficient gelation under mild, aqueous, and near-neutral conditions, as well as the sensitivity of the cross-linking to temperature and pH. These theoretical insights align closely with the observed pH-dependent rheology, wherein acidic conditions strengthen the network by promoting hydroxyl–boron coordination, and with the reversible thermal transitions that modulate network integrity. Among the tested formulations, SC/PNB10 = 5/5_7.5 wt % emerged as the optimal composition, combining high storage modulus, rapid and reversible sol–gel transitions, robust self-healing, and excellent injectability. The ability to fine-tune gelation temperature, mechanical properties, and recovery behavior simply by adjusting copolymer composition, SC/PNB ratio, and solid content—without altering processing conditions—highlights the versatility of this design. Furthermore, the sol state at low temperature and gelation at physiological temperature, together with the affinity of boronate ester bonds for cell membranes, position SC/PNB hydrogels as promising candidates for injectable drug delivery systems and 3D cell culture scaffolds, where controlled encapsulation, on-demand release, and postculture cell recovery are desired. In summary, by integrating rheological characterization with DFT-based molecular simulations, this work elucidates the gelation mechanism of boronate ester hydrogels from the molecular to the macroscopic scale and introduces a temperature-sensitive, triple-staged sol–gel–sol platform with substantial promise for advanced biomedical applications. Despite the promising thermoresponsive and self-healing properties demonstrated in this work, several challenges remain for future biomedical translation. These include the need for a deeper understanding of the effects of molecular parameters and improved evaluation of stability under diverse conditions, including proteins, salts, and reactive species. Future studies will also focus on in vitro/in vivo biocompatibility, controlled drug release performance, and optimization of the transition temperature window for specific biomedical applications.

## Supplementary Material



## Notes

Phase transition temperature analysis of PNBs; gelation optimization; rheology analysis and self-healing behaviors; injectability; and degradation of SC/PNB hydrogels (PDF)
