# Supplementary material for: Molecular Simulation-Elucidated Boronate Ester Gelation in Succinyl Chitosan-Based Hydrogels with Triple-Staged Thermoresponsive Sol–Gel–Sol Behavior
Source: Biomacromolecules. 2026 Jun 22;27(7):4499–510. doi: 10.1021/acs.biomac.6c00414 (PMC13370782; doi:10.1021/acs.biomac.6c00414)
Supplement: Supplementary file 1 [file bm6c00414_si_001.pdf]

Supporting Information

Molecular Simulation–Elucidated Boronate Ester Gelation in Succinyl Chitosan-Based Hydrogels with Triple-Staged Thermo-Responsive Sol–Gel–Sol Behavior

Wen-Hsin Wang[1]†, Yue-Ci Wu[1]†, Hsiu-Min Hung[1], Kai-Chun Wang[2], Dhayanithi Senthilkumar[1,3], Ying-Chieh Hung[1*], Chih-Yu Kuo[1,2,3,4*]

[1]Department of Chemical Engineering and Biotechnology, National Taipei University of Technology, Taipei City 10608, Taiwan.

[2]Institute of Biochemical and Biomedical Engineering, National Taipei University of Technology, Taipei City 10608, Taiwan.

[3]International Graduate Program of Energy and Optoelectronic Materials Program (EOMP), National Taipei University of Technology, Taipei City 10608, Taiwan.

[4]High-value Biomaterials Research and Commercialization Center, National Taipei University of Technology, Taipei City 10608, Taiwan.

†stands for equal contribution

[*]Corresponding authors: **Chih-Yu Kuo,** e-mail: chihyukuo@ntut.edu.tw

Ying-Chieh Hung, email: hungyc@ntut.edu.tw

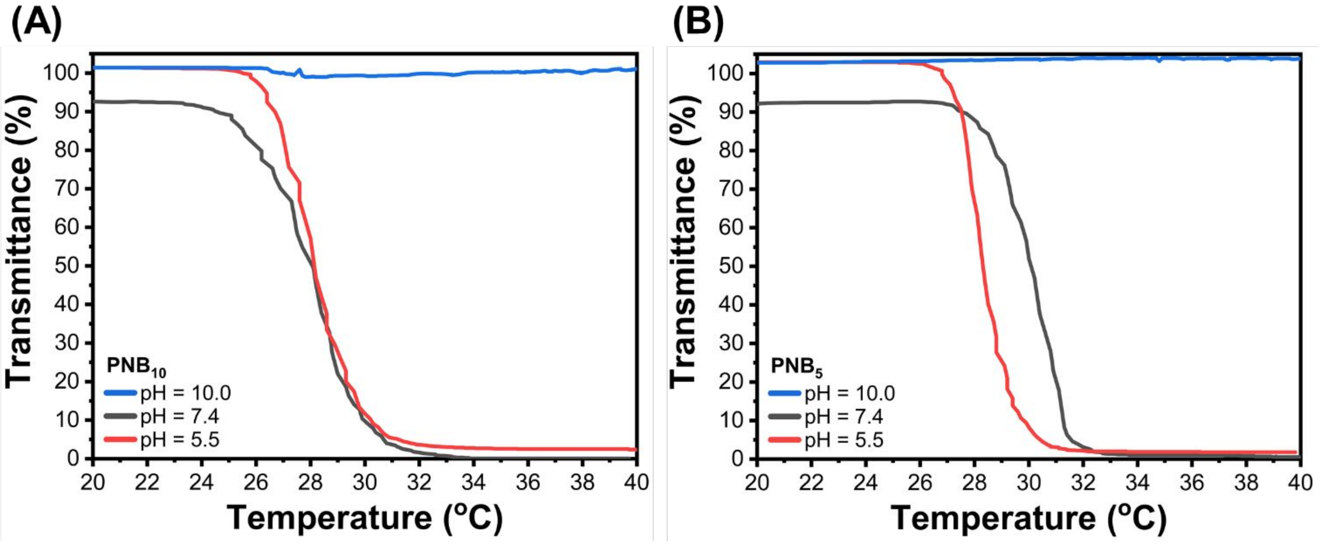

Figure S1. The phase transition temperature analysis of PNBs at various pH conditions. (A) PNB_{10}, (B) PNB_5.

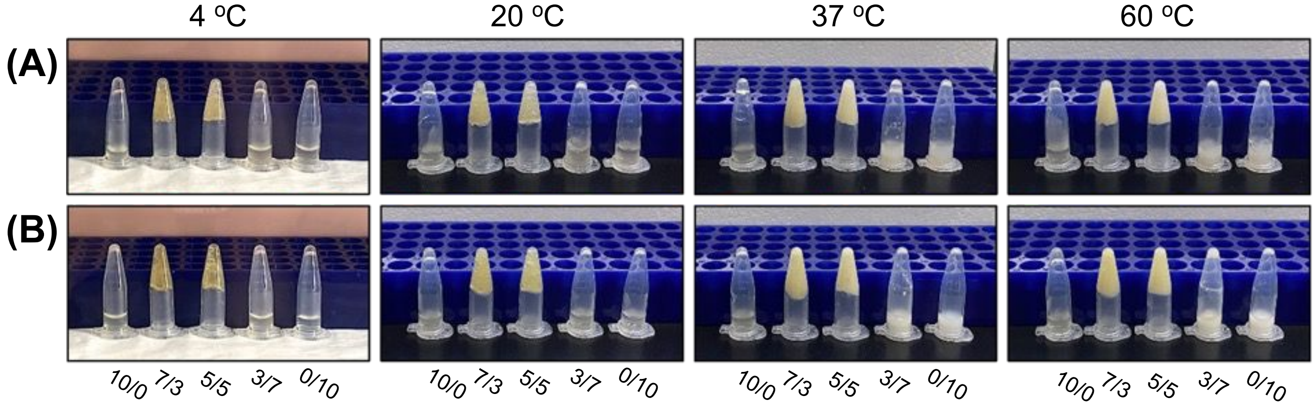

Figure S2. Optimization of the SC/PNB hydrogels compositions at 10 wt% with various SC/PNB weight ratios under pH = 7.4 (A) SC/PNB_{10}, (B) SC/PNB_5 under different temperatures.

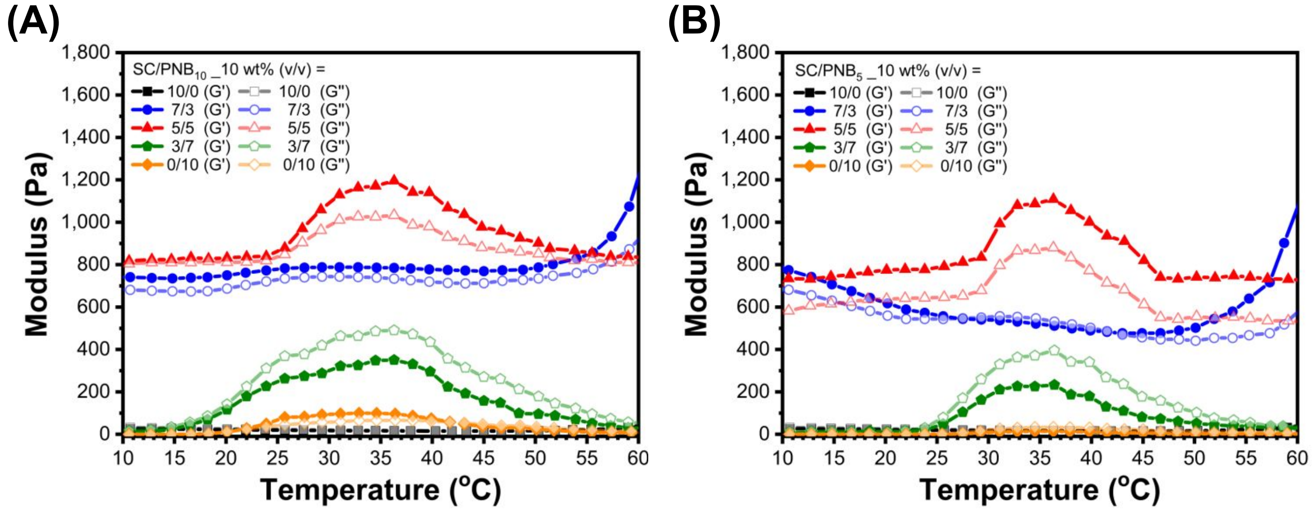

Figure S3. Rheology analysis for SC/PNB solutions at constant strain and frequency (γ = 1%, f = 1 Hz) from 10 °C to 60 °C with the heating rate of 3 °C/min. (A) SC/PNB$_{10}$_10 wt%; (B) SC/PNB$_5$_10 wt%.

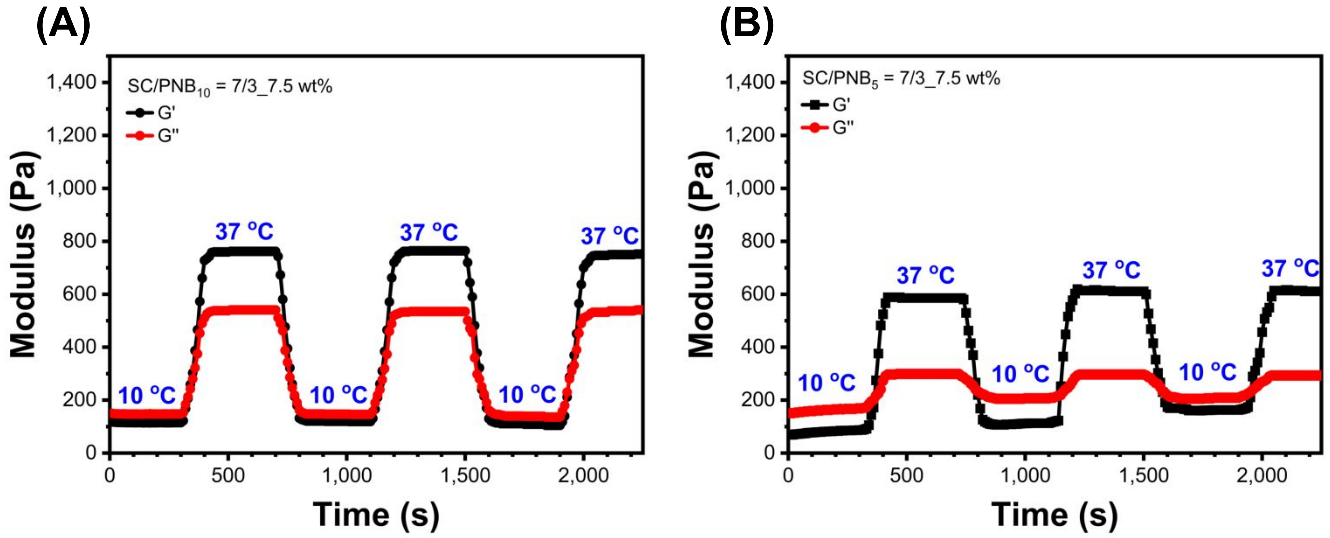

Figure S4. Cyclic temperature sweeping (10 °C or 37 °C) of SC/PNB = 7/3_7.5 wt% solutions at constant strain and frequency (γ = 1%, f = 1 Hz) (A) SC/PNB$_{10}$; (B) SC/PNB$_5$.

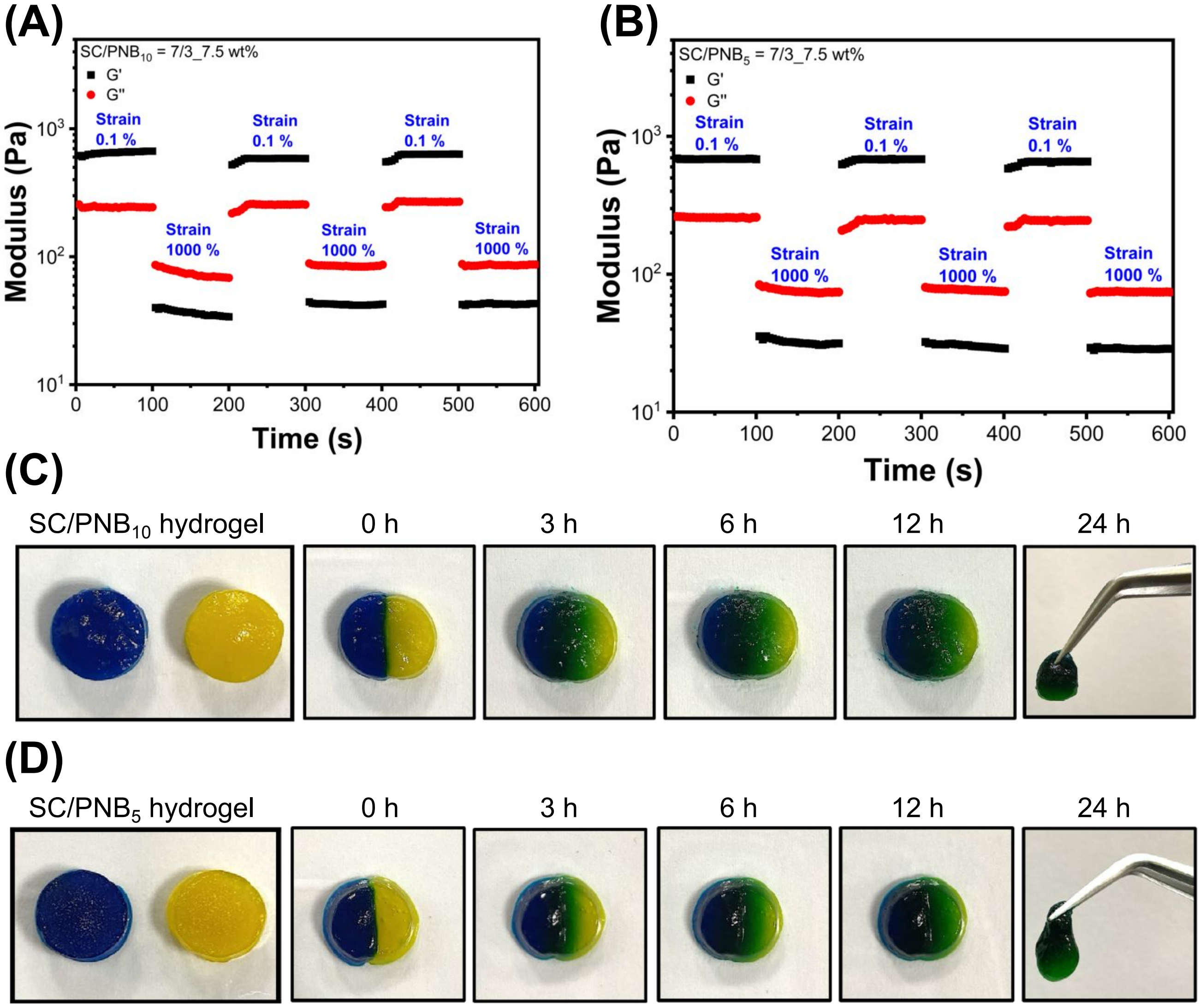

Figure S5. Self-healing behavior of SC/PNB = 7/3_7.5 wt% hydrogels at 37 °C. Dynamic strain measurement (γ = 0.1% or 1000%): (A) SC/PNB$_{10}$ hydrogel; B) SC/PNB$_5$ hydrogel. Macroscopic self-healing images of (C) SC/PNB$_{10}$ hydrogel; (D) SC/PNB$_5$ hydrogel.

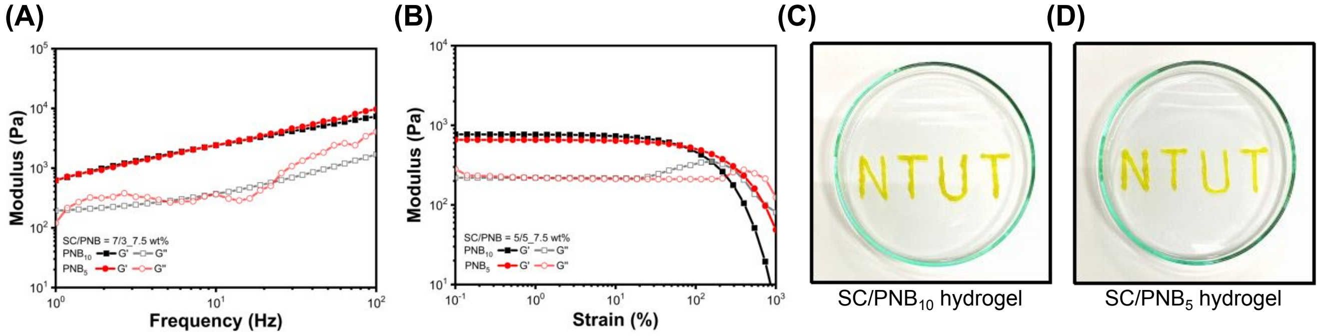

Figure S6. Rheology analysis for SC/PNB = 7/3_7.5 wt% hydrogels at 37 °C with PNB_{10} or PNB_5. (A) frequency sweeps from 1 to 100 Hz with a constant strain of 1% (B) strain sweep scanning ranging from 0.1 % to 1000 % at a fixed frequency of 1 Hz. Injectability of SC/PNB= 7/3_7.5 wt% hydrogels. (C) SC/PNB_{10}_7.5 wt% hydrogel; (D) SC/PNB_5_7.5 wt% hydrogel.

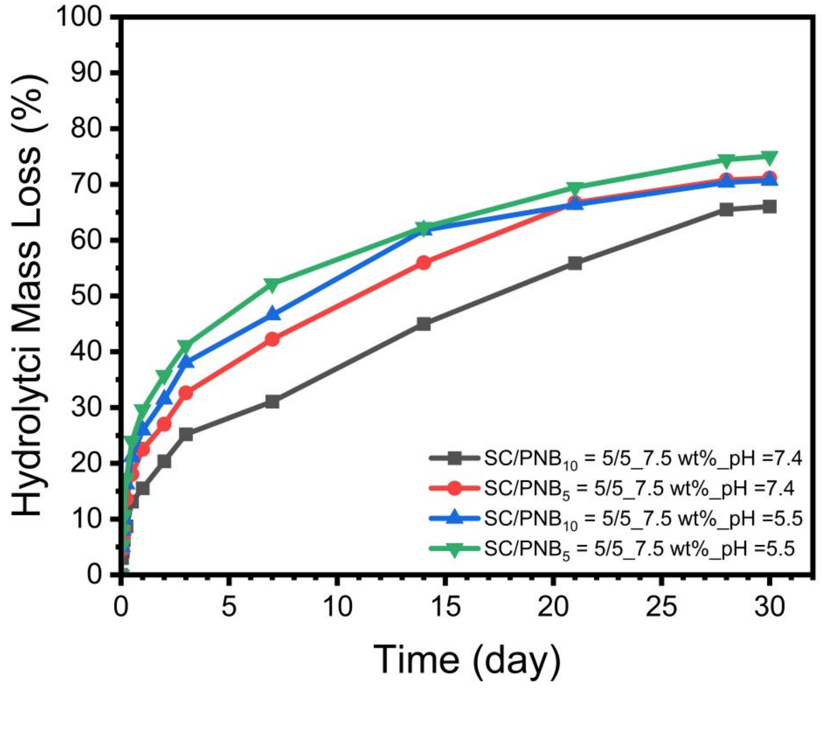

Figure S7. The pH-responsive hydrolytic mass loss behaviors of SC/PNB = 5/5_7.5 wt% hydrogels under various pH conditions.
